# Determinants and constraints of carrot (*Daucus carota* L.) production and marketing in Cameroon

**DOI:** 10.1371/journal.pone.0296418

**Published:** 2024-01-05

**Authors:** Maxime Merlin Tonfack Djoufack, Eric Bertrand Kouam, Edith Marius Kouam Foko, Mariette Anoumaa, Gilles Raoul Lontsi Meli, Pierre Marie Kaktcham, François Ngoufack Zambou

**Affiliations:** 1 Department of Biochemistry, Faculty of Science ‐ University of Dschang, Dschang, Cameroon; 2 Department of Crop Science, Faculty of Agronomy and Agricultural Science, University of Dschang, Dschang, Cameroon; 3 Department of Physiological Sciences and Biochemistry, Faculty of Medicine and Pharmaceutical Sciences, University of Dschang, Dschang, Cameroon; 4 Department of Plant Biology, Faculty of Science, University of Dschang, Dschang, Cameroon; 5 Department of Soil Science, Faculty of Agronomy and Agricultural Science, University of Dschang, Dschang, Cameroon; West Bengal State University, INDIA

## Abstract

The market gardening sector in Cameroonian agriculture is facing a challenge in producing vegetables to meet consumer needs. Among these vegetables, carrot is known to play an important role in the livelihood of millions of people around the world. It is therefore important to understand its technical itinerary in the different agricultural basins in order to provide further information on carrot production. This study aimed to identify the different production constraints faced by carrot growers by providing information on applied growing systems. Two surveys were conducted using a questionnaire simultaneously with carrot traders and producers. A total of 218 carrot traders and 62 producers were interviewed. Nine basins were identified as main carrot production zones in Cameroon. In the production basins, five main varieties namely *New Kuroda*, *Pamela+*, *Madona*, *Amazonia and Vanessa F1* were identified. The agricultural yield of carrots is related to the growing area, fertilization method, size of the field and ploughing. Farmers use an integrated fertilization approach based on chemical fertilizer (N-P-K: 20-10-10) and chicken manure at various doses, from 250 to 500 Kg.ha^-1^ and 2.5 to 9 t.ha^-1^ respectively. Factors such as farmland area, number of cultivated plots, experience in cultivation, family size and amounts of organic and chemical fertilizers used have been found to have significant impact on carrot production. However, among the many technical problems faced by producers and which result in low carrots yields, is the lack of knowledge of soil properties. This in turn contributes to inappropriate fertilization and poor choice of the appropriate variety to be cultivated. Low germination, the lack of efficient irrigation systems and the high costs of agricultural inputs are the main constraints that affect carrot production. Although valued by market gardeners, the benefit in a production season is not always enough to encourage more farmers to grow carrots. Thus, there is a need to develop a follow-up policy for the quality and high yield production of the carrot sector.

## 1. Introduction

In Cameroon, as in many other Sub-Saharan African (SSA) countries, vegetables are not widely consumed in terms of quantity. Kamga et al. [1] estimated that, only 46 grams of vegetables are consumed per capita per day in Cameroon. This represents only 11.5% of the FAO/WHO recommended intake. Low consumption of vegetables in Cameroon is associated with low production and unavailability and/or accessibility to all segments of the population. Moreover, low-cost vegetables (carrots, celery, parsley, leeks, green beans, basil, etc.) that can be accessible to all, are not promoted on a large scale and are therefore not considered in the statistical evaluation of agricultural products in Cameroon. Indeed, improving the production and consumption of vegetables could be considered as the most direct and least expensive way for poor urban and rural populations to increase the micronutrients in their diet [2]. This could greatly contribute to reduce the risk of nutrition-related non-communicable diseases. Among these vegetables, carrot (*Daucus carota* L. fam Apiaceae) is one of the most nutritious root vegetables [3].

Carrot (*Daucus carota* L.) is a biennial herbaceous species of the Apiaceae family and is considered one of the most important root vegetables in the world [4]. It is cultivated worldwide and especially in temperate regions. Although there are 60 species of the genus *Daucus* that are wild varieties, carrot (*Daucus carota*, the domesticated form) is the most cultivated and originates from Central Asia [5]. The flesh colour can be orange, yellow, white, red, purple or even black. However, the colour and size depend on the variety grown [6]. The domesticated carrot (*Daucus carota* L. ssp. *sativus*) can be separated into two genetically distinct groups: Eastern (Asian) and Western (European and American) carrots. Carrots are 10^th^ in terms of nutritional value [7]. They are increasingly consumed due to their richness in carotenoids (provitamin A) [8] which are converted to vitamin A in the human body and are important for eye health [9]. Furthermore, carrots also contain appreciable levels of several other functional components and therefore have a beneficial influence on human health. They are a good source of carbohydrates, minerals (Ca, Fe, Na, K, Mg, Cu and Zn), carotenes [10] and vitamins (thiamine, riboflavin, niacin and vitamin C) [11, 12]. It has been shown that, in addition to the important nutritional value of carrots, the trend in antioxidant activity of the varieties is similar to their nutritional composition [13].

Carrot production, mainly based on orange-colored varieties, has quadrupled over the past 45 years to more than 34,17 t.ha^-1^ worldwide, making carrots one of the 10 most economically important vegetable crops worldwide [14, 15]. China, USA, Uzbekistan and Poland producing 60% of the world’s carrots, are the main carrot producing countries [15]. The first carrot producing country in Africa (14749 ha) with a production rate of 32.38 t.ha^-1^ is Morocco. Indeed, carrot cultivation is widespread in Algeria, Niger, Senegal, Cameroon and several other African countries [16]. Despite the very low yield produced, Cameroon exports carrots to Gabon and Equatorial Guinea [16]. Current studies on carrots aim to improve the plant’s growth potential and the selection of varieties with good root quality. In this regard, [17] studied the influence of chicken manure and nitrogen, phosphorus, and potassium (NPK) fertilizer (17-17-17) on carrot growth and yield in Rwanda and reported that the combination of chicken manure and NPK showed a significant influence on carrot growth and yield. Studies by [18] in Northern Nigeria showed that application of nitrogen, phosphorus and farmyard manure significantly increased agronomic parameters. However, because there are no exact recommendations for the use of organic fertilizers [19] and the application of inorganic fertilizers depends on soil properties, farmers are unaware of the specific requirements of this crop and of new production methods to increase their yield. This often leads them to engage in erroneous use of fertilizers [20]. In order to shed some light on the limitations faced by carrot farmers and to highlight the different factors that hinder high yields, several studies have been conducted. These studies have looked at the characteristics that affect production and consumption of carrots. Among those factors, it has been reported that training farmers on healthy and environmentally friendly farming practices increases productivity and results in healthy production [21]. Farmers’ access to manure and farm tools and the increased participation of women in vegetable cultivation, could yield huge benefits in terms of efficiency of vegetable production in Cameroon [22]. According to [23], there is a significant positive relationship between vegetable farming and farmers’ livelihoods. Moreover, many constraints (poor market management and lack of irrigation facilities) and challenges such as disorderly urban growth, price fluctuation and vegetable diseases negatively affected profitability. In Cameroon, work on carrots have been focused on determining household consumption frequencies, the influences of fertilizer types and carrot varieties on production yields and nutritional and organoleptic qualities [1, 10, 24, 25]. These studies did not take into account the factors that govern carrot cropping systems and that are applied by farmers who deliver carrots to markets for consumption. In addition, the constraints faced by these growers that hinder intensive production are not widely known. It would therefore be interesting to have more information on these aspects of carrot production in Cameroon.

However, as the consumption and marketing of carrots is growing exponentially worldwide due to their attractive sensory properties and richness in carotenoids [8, 26], the contrast is that consumers are looking for healthy carrots while carrot growers are looking for the benefits. As such, the production of carrots becomes a general and particular concern for all segments of the population. Therefore, the identification of the constraints to the intensive production and marketing of carrots of good nutritional and commercial quality for large-scale consumption by the population would be essential for the welfare of consumers. The objective of the present work was therefore to assess the knowledge levels of traders on carrot cultivation and its production constraints in Cameroon, in order to improve our knowledge on the factors and constraints affecting carrot production in Cameroon. We hope that the results from the present study will allow the design of follow-up policies aiming at improving the quality and the yield of carrots production in Cameroon.

## 2. Materials and methods

The present study was conducted in the major carrot producing zones of Cameroon. Among the five agro-ecological zones of Cameroon, a selection of zones confirmed as carrot production basins was made after a preliminary survey.

### 2.1. Description of the study area

**Fig 1:**
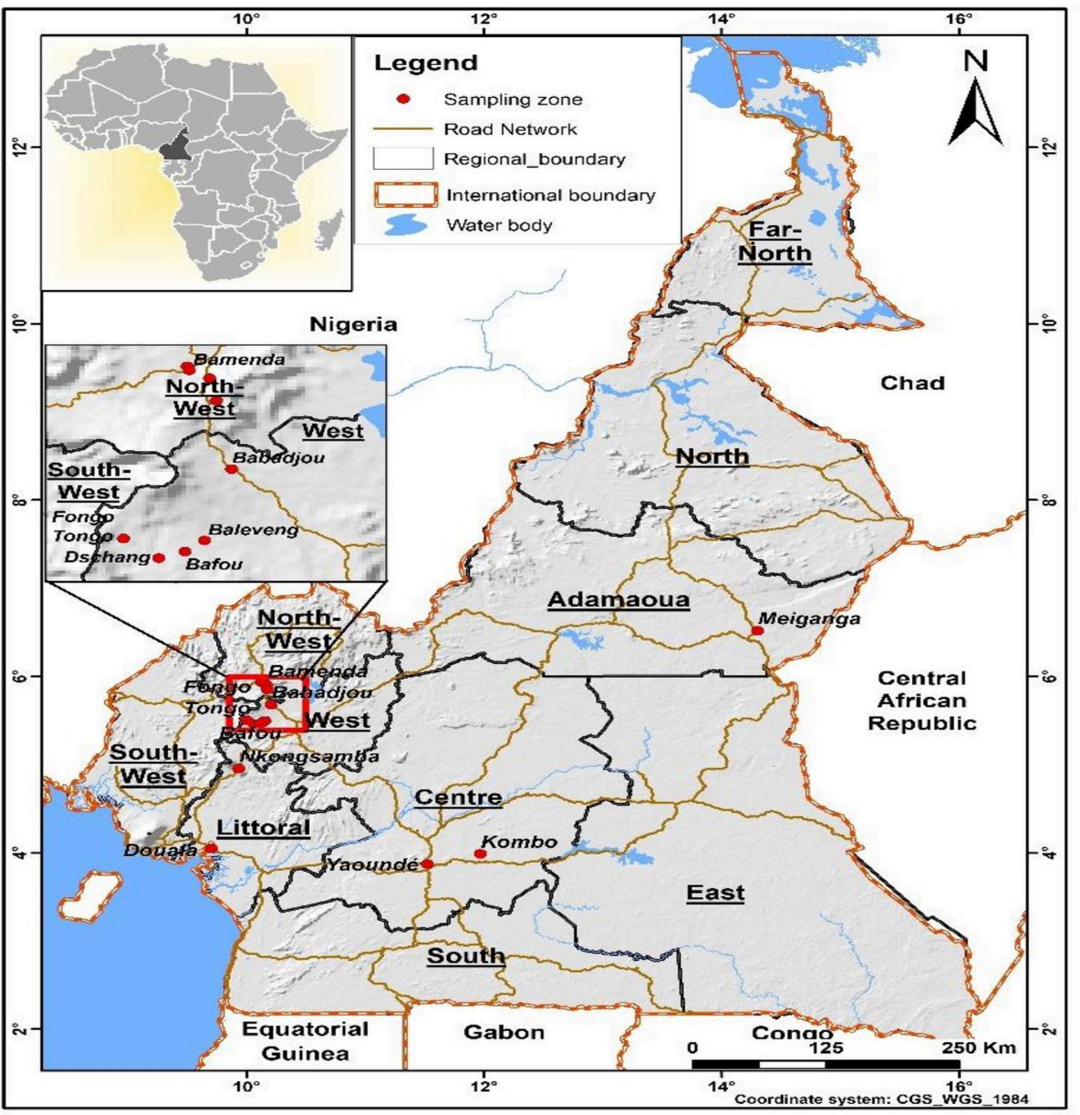
Map of the different data collection zones.

Cameroon is located on the Gulf of Guinea at the intersection of West and Central Africa. With an area of 475,650 km², it extends from 2° to 13° North latitude and 9° to 16° East longitude from Greenwich (at its widest point 800 Km) [27]. It is triangular in shape and shares borders to the West with Nigeria, to the North with Chad, to the East with the Central African Republic, to the South with Congo, Gabon and Equatorial Guinea, and a 400 Km strip of the Atlantic Ocean to the Southwest [28]. Several types of natural environment contribute to the country’s geographical diversity. Across the ten (10) regions of the country, there are five (05) distinct agro-ecological zones with different rainfall and soil characteristics. All these five zones were initially selected as survey sites until the elimination of the zones declared as non-carrot producing basin after a preliminary survey made among farmers and traders of these regions of the country. As a result, three (03) agro-ecological zones were selected for this study. These include the Western Highlands (West and North-West regions), which are rich in volcanic soils; the humid forests with monomodal and the humid forests with bimodal rainfall (Centre, East, Littoral, South and South-West regions), which are characterized by dense vegetation, a vast hydrographic network, and a hot and humid climate with abundant rainfall [29]. The different data collection areas are shown in Fig 1.

### 2.2. Data collection strategy

The socio-economic analysis consisted in identifying the carrot varieties grown in Cameroon, the types and doses of fertilizers used by carrot growers and the average production frequency in relation to the areas cultivated. For this purpose, a survey was conducted in carrot production basins of each agro-ecological zone where carrots are predominantly grown. Another survey was first carried out in the main vegetable urban markets of Douala, Yaoundé and Dschang. This was done in order to identify the preference criteria often guiding the traders in the choice of the carrot production site. This survey also aimed at identifying the different types of products accompanying the sale of these carrots and at evaluating their level of knowledge about carrot cultivation. Access to the field sites was approved by Mr. Denis AMIGUIM, who is the Regional Delegate for agriculture for the Western region of Cameroon.

Data collection was done in chronological phases in order to ensure the quality of the data at the end of the survey. Thus, an inventory was made on both the different carrot seeds found on Cameroonian markets, and the types of inorganic and organic fertilizers frequently used. The inventory of carrot seeds was done by asking vendors about the availability of the different types of seeds they had in their shops and information on the different types of fertilizers commonly used was obtained from the farmers. It should be noted that a mapping of the most popular cities for the sale of carrots was made in order to serve as a reference for the survey.

#### 2.2.1. Assessment of marketing channels and constraints

A total of two hundred and eighteen (218) traders were surveyed in Yaoundé, Douala and Dschang markets. The rationale for choosing the cities of Yaoundé and Douala is based on their cosmopolitan populations and the high purchasing power of vegetables among their populations. The survey was semi-administered and was conducted with traders who had agreed to answer all the questions. The questions were grouped into two sections. Section one was designed to collect socio-demographic information of the respondents, while section two was designed to collect information on the traders’ motivations to sell carrot, their level of knowledge about the carrot production areas for the purpose of guiding our surveys in the production sites and the variety of carrots delivered to them. The survey was conducted during the period of August ‐ September 2019. Data collection was done at all the main carrot outlets in each surveyed site. Therefore, markets serving as supply points for all traders in the different study towns were considered as the surveyed sites.

#### 2.2.2. Assessment of carrot production constraints

A total of sixty-two (62) farmers were surveyed in the carrot production basins of Cameroon during the periods of October ‐ November 2019 and February ‐ March 2020. Those periods corresponded to the peak periods for carrot production. This survey was conducted with farmers who had consented to answer the questions and the survey type was semi-administered. The questions were grouped into three sections. Section one was based on the socio-demographic data of the respondents; section two was based on data related to indicators of carrot productivity; and section three focused on the productivity and profitability of carrot farming in Cameroon.

In each village, interviews were conducted with the help of a local guide/translator to facilitate discussions and exchanges with farmers. Field observations were complementary to the questionnaires.

#### 2.2.3. Selection of potential factors related to the carrot production route

A total of 35 variables were selected to assess the different factors affecting carrot production in Cameroon as well as the relationship between these variables and the livelihoods of carrot farmers. Each variable was selected according to the socio-demographic, economic and carrot production routes used by farmers in order to understand the knowledge, reasons for selecting varieties and cultivation practices employed by carrot farmers. Each variable was selected on the basis of hypotheses developed from a pre-test of the questionnaire with a few farmers at one of the survey sites. All data were collected through surveys in the regions considered as carrot production basins in Cameroon. A description of each selected variable is given in Table 1.

**Table 1:** Variables selected for analysis using the non-parametric bivariate correlation test and their descriptions.

Variables	Description
Growing region	The geographical location of the growing area highlights the different carrot production basins and provides information on the typology of the cultivated land which has an influence on production
Region of origin	Farmers from regions with farming practices as their main activity have experience in growing vegetables
Gender	Work potential and male advantage in agricultural knowledge and networks
Age	Proxy for agricultural experience
Religion	Religious affiliation or belief affects the number of working days farmers can do while growing this vegetable
Education	Literate farmers have a better understanding of production and marketing
Size family	Smaller family size indicates lower expenditure and higher savings potential
	A larger family means a larger pool of labour to work with on the farm
Training on vegetable growing	Trained farmers have advantages in growing vegetables
Size of the workforce	The number of people involved in field work influences the speed of land use
Local market	Access to local markets increases the likelihood of selling market garden products
Type of farmland	Irrigated flat land is more productive than sloping land
Mode of tenure (Ownership)	The amount paid by tenants for land increases by 10% each year
Size/area of farmland	Increased size of farmland can lead to increased production and income
Number of plots of land	The number of plots of land farmed provides information on the extent of the farmers’ area of operation and their production
Type of workforce	The nature of the labour force used affects profits
Year the farmer started growing vegetables	An earlier start date indicates greater experience and more efficient production
Loans and grants	Loans and grants ease the financial burden on farmers
Use of manure	Organic vegetables are more valuable and manure replaces chemical fertilizers
Use of chemical fertilizers	The use of chemical fertilizers increases production
Farmer’s profession	The daily occupations of the farmers provide information on the time they spend on carrot cultivation and the sources of investment
Nature of carrot seed	The seed varieties used by farmers provide information on the types of varieties most favoured by growers
Varietal choice	The selection of carrot varieties made by farmers highlights the advantages of growing carrots and the types of varieties most commonly sold on Cameroonian markets
Origin of seed varieties	The source of the carrot varieties used provides information on the existence or not of local carrot varieties
Production frequencies per plot	The production frequency per plot provides information on the number of times a plot is farmed and the annual profitability
Crop rotation	The process of crop rotation helps to manage the notion of saving soil nutrients
Land fertility management	Good soil fertility management limits the expense of purchasing fertilizers for pre-cultivation soil improvement and productivity efficiency
Amount of manure used	The manure rates applied by the farmers provide information on the average rates applied during carrot cultivation
Amount of chemical fertilizer used	The doses of chemical fertilizers applied by the farmers provide information on the average doses and different formulations of fertilizers applied during carrot cultivation
Average production yield per plot	Farmers’ income levels increase with the number of plots of land farmed and the type of fertilizer applied
Production rate	Income levels increase when production is year-round
Production constraints	Poor management of factors that hinder productivity leads to lower incomes
Marketing constraints	Poor choice of farming location, seed varieties and growing season influence the sale of carrots
Selling price of carrot fillet	Carrot production periods affect the carrot trade
Good growing season	Rainy seasons make it easier to grow carrots without setting up an irrigation system
	The sunny seasons make it difficult to produce carrots on a large scale due to water scarcity and require an adapted irrigation system

### 2.3. Analysis of the data collected

The data from the trader questionnaires were analyzed using Epi Info version 3.2. to determine the personal motivations of carrot sellers in Cameroon and to assess their level of knowledge about the areas of origin of the carrots and the carrot varieties they sell. Descriptive analysis was performed to provide frequencies of the demographic characteristics of the respondents as well as the frequencies of the different variables relating to the level of knowledge of the traders about cultivating carrots.

The analysis of the farmer survey data combined qualitative and quantitative analysis tools. Data from the completed questionnaires were entered and coded in Microsoft Excel and then exported to Statistical Package for Social Sciences (SPSS) (version 22 developed by International Business Machine (Armonk, NY, USA)) for further analysis. The data analysis involved the use of descriptive statistics, including frequencies to describe the socio-economic characteristics of the farmers. The Microsoft Excel software (version 2013) was also used to produce the figures showing the types of carrot varieties most commonly grown and the reasons why those varieties are grown. The Chi-square test at the 5% probability level was used to determine the independence or dependence (correlation) between the different variables affecting the carrot production system. Variables with a probability threshold below 5% were significantly associated with each other, while those with values above 5% were not significantly associated.

## 3. Results

### 3.1. Socio-demographic characteristics and level of knowledge of traders on carrot cultivation in Cameroon

#### 3.1.1. Socio-demographic characteristics of traders

Table 2 presents the socio-economic characteristics of the carrot traders. This table shows that most of the carrot traders are from the West Region (78.44%), followed by the Central region (13.75%); there is a Congolese trader (0.46%) who also sells carrots in Cameroon. Out of the 218 surveyed traders, 75.69% are women whereas 24.31% are men. Most of them attained secondary education level (72.02%). Very few traders attained higher education level (6.88%). These carrot traders are in the 30 to 45 years old age group (62.84%).

**Table 2:** Socio-demographic characteristics of carrot traders.

Characteristics	Attributes	Size	Percentage (%)
Region of origin	Centre	30	13.75
	East	2	0.92
	Far North	1	0.46
	Kassaye	1	0.46
	Littoral	2	0.92
	North West	11	5.05
	West	171	78.44
Gender	Male	53	24.31
	Female	165	75.69
Education level	Primary	46	21.10
	Secondary	157	72.02
	University	15	6.88
Age (years)	[15 ‐ 30]	16	7.34
	[30 ‐ 45]	137	62.84
	[45–60]	56	25.69
	[60–75]	9	4.13

#### 3.1.2. Traders knowledge about carrots

Table 3 shows factors that determine the level of knowledge of carrot sellers about the different methods of carrot cultivation. It appears from this table that, out of the 218 vendors surveyed, 199 (91.28%) declared that they knew the carrot production areas. Of the 13 carrot production areas surveyed, Dschang (74.87%), Bamenda (68.84%), Babadjou (49.74%), Bafou (21.60%), Santa (16.58%) and Bamock (13.56%) were the best known. Of the 199 vendors who knew the carrot production basins, 134 (67.68%) said they had preferences for certain basins. Of the 06 preferred production areas, only those of Bamenda (59.70%), Dschang (32.83%), Santa (11.19%) and Bafou (9.70%) were the most preferred because of the large size and sweetness of the roots. It appears that traders are also interested in the length (47.14%) and the deep orange colour (35.71%) of the carrot roots. Of the 64 sellers who did not mention preferences for carrot production areas, 56.80% were unaware of the specific characteristics of carrots from different production areas and 25.75% of sellers buy according to market prices. With regard to the names of carrot varieties, 47 sellers (21.56%) stated that they had knowledge of certain variety names. Of the 08 varieties surveyed in the questionnaire, *Pamela+* (70.21%) and *New Kuroda* (17.02) are the best known, and we recorded 40.42% as additional varieties (*Myranda*, *Vikima*, *Vilmorin*, *Juliette*, *Roma and Tesier)* listed by sellers. It is also evident from Table 3 that the majority of the vendors have been trading carrots for more than 10 years (38.08%) and they reported selling carrots for profit (52.29%) and also for subsistence reasons (76.14%). However, carrot sellers face marketing problems such as rotting (55.04%), high costs (55.04%) and other constraints like morphological defect, inadequate filling of bags, poor storage, and rodents (11.46%).

**Table 3:** Traders’ knowledge of carrot cultivation.

Characteristics	Attributes	Size	Percentage (%)
Knowledge of production basins	Yes	199	91.28
	No	19	8.72
Carrot production basins	Babadjou	99	49.74
	Bafou	43	21.60
	Baleveng	1	0.50
	Baloum	3	1.51
	Bamenda	137	68.84
	Bamendou	5	2.51
	Bamock	27	13.56
	Bertoua	2	1.00
	Dschang	149	74.87
	Fongo-Tongo	2	1.00
	Foumbot	17	8.54
	Maroua	1	0.50
	Santa	33	16.58
Preference of production basins	Yes	134	67.68
	No	64	32.32
Preferred production basins	Babadjou	4	2.98
	Bafou	13	9.70
	Bamenda	80	59.70
	Bamock	7	5.22
	Dschang	44	32.83
	Fongo-tongo	1	0.74
	Foumbot	1	0.74
	Santa	15	11.19
Preference criteria	Large root size	64	39.76
	Sweet carrots	61	37.89
	Better shelf life	22	13.66
	Availability	14	8.69
Other preference criteria	Accessibility	3	4.28
	Good morphological quality	33	47.14
	Deep orange colour	25	35.71
	Satisfactory filling of the bags	14	20.00
Non-preference criteria	Self-supply	1	1.42
	Single source of supply	11	16.67
	Price dependent	17	25.75
	Self-generation	1	1.51
	Ignorance of differences	37	56.06
Knowledge of varieties delivered	Yes	47	21.56
	No	171	78.44
Delivered varieties	*Pamela+*	33	70.21
	*New Kuroda*	8	17.02
	*Amazonia*	3	6.38
	*Sakata*	3	6.38
	*Pamela*	1	2.12
	*Bahia*	1	2.12
	*Shakira*	1	2.12
	Others varieties	19	40.42
Experience carrot trader (years)	1	5	2.29
	2	23	10.55
	4	30	13.76
	5–6	39	17.89
	7–8	20	9.17
	8–10	18	8.26
	>10	83	38.08
Proofs of sale	Beneficial	114	52.29
	High demand	36	16.51
	Livelihood	166	76.14
Marketing constraints	Rotting	120	55.04
	High cost	120	55.04
	Commercial space	18	8.26
	Insufficient	14	6.42
	High cost of transport	10	4.58
	Others constraints	25	11.46

### 3.2. Socio-demographic characteristics of carrot producers

Details of the socio-demographic characteristics of the carrot producers surveyed are presented in Table 4. It appears from this table that carrot growers in Cameroon are made up of both genders with a predominance of males (75.8%) compared to females (24.20%). They are between [35–45] years old (41.9%) and [45–55] years old (37.2%). The majority of producers reported being married in a monogamous regime (87.1%) compared to married in a polygamous regime (4.8%) and single (8.1%). The results also showed that most of these producers attained the secondary education level (62.9%) meanwhile only 19.40% of them attained higher education level. Very few farmers dropped out at the primary school (17.7%). Irrespective of the level of education of the farmers, 43.5% of them have received vocational training in crop production. The majority of farmers are Christians (82.2%) whereas some few are animists (17.7%). Family size is 6–10 members for 37.1% and 11–15 members for 45.2% of respondents.

**Table 4:** Socio-demographic characteristics of carrot producers.

Characteristics	Frequencies (N = 62)	Percentage (%)
**Age (years)**		
[25 ‐ 35]	08	12.80
[35 ‐ 45]	26	41.90
[45–55]	23	37.20
[55–65]	05	8.10
**Gender**		
Male	47	75.80
Female	15	24.20
**Marital status**		
Monogamous Married	54	87.10
Polygamous married	3	4.80
Single	5	8.10
**Education level**		
Primary	11	17.70
Secondary	39	62.90
Higher	12	19.40
**Religious obedience**		
Christian	51	82.20
Islamic	0	0
Pagan	3	4.80
Traditional	8	12.90
**Family size**		
1	1	1.60
2–5	4	6.50
6–10	23	37.10
11–15	28	45.20
≥16	6	9.70
**Agricultural training**		
Yes	27	43.50
No	35	56.50

### 3.3. Characteristics of carrot growers

The different characteristics of carrot producers are presented in Table 5. In this table it can be observed that the majority (67.70%) of the producers are from the West region of Cameroon followed by those from the Southwest region (30.60%) and the Far North (1.6%). In addition, the Bafou area (41.90%) was recorded as the largest carrot production basin in the Western Region while Nkongsamba (30.6%) was the largest production basin in the Littoral Region. The size of the exploited land vary from 1 to 3 hectares for the majority of respondents (66.10%) and from 4 to 6 hectares for 12.90% of them. These farmers reported owning (75.80%), renting (53.20%) and receiving as gifts (22.60%) the areas of land they cultivate. In most cases, the land is subdivided into 3 to 4 (56.50%) or 5 to 6 (33.90%) plots of land, with the majority of the workforce being volunteers (85.50%) and family members (83.90%). It is important to underline the fact that in addition to farming, these carrot producers have other sources of income, trade being the most cited activity (56.50%). In fact it represents the main farming funding source for 71.00% of farmers involved; unlike other producers (27.40%) who resort to loans. Very few respondents (1.60%) benefit from the attention of development aid program. Experience being an important factor in increasing the productivity of crops and which is acquired only with time, most carrot producers in Cameroon stated that they had been growing carrots for 4 to 6 years (32.30%), 7 to 9 years (29.00%) and 10 to 15 years (22.60%).

**Table 5:** Characteristics of carrot growers.

Characteristics	Frequencies (N = 62)	Percentage (%)
Region of origin		
Far North	1	1.60
West	42	67.70
South West	19	30.60
Growing zone		
Babadjou	6	9.70
Bafou	26	41.90
Baleveng	1	1.60
Dschang	2	3.20
Fongo-Tongo	3	4.80
Kombo	2	3.20
Meiganga	3	4.80
Nkongsamba	19	30.60
Mode of tenure (ownership)		
Owner	47	75.80
Tenant	33	53.20
Donation	14	22.60
Land area (ha)		
≤1	11	17.70
1–3	41	66.10
4–6	8	12.90
7–9	2	3.20
Number of plots on the holding		
1–2	1	1.60
3–4	35	56.50
5–6	21	33.90
7–8	4	6.50
≥9	1	1.60
Type of workforce		
Family	52	83.90
Salary	5	8.10
Mutual aid	9	14.50
Voluntary work	53	85.50
Parallel profession		
Trader	35	56.50
Public sector employee	5	8.10
Private sector employee	9	14.50
Craftsperson	5	8.10
None	8	12.90
Funding source		
Loan-sharking	17	27.40
Program	1	1.60
None	44	71.00
Experience (years)		
≤2	2	3.20
4–6	20	32.30
7–9	18	29.00
10–15	14	22.60
≥16	8	12.90

### 3.4. Carrot production system in Cameroon

**Fig 2:**
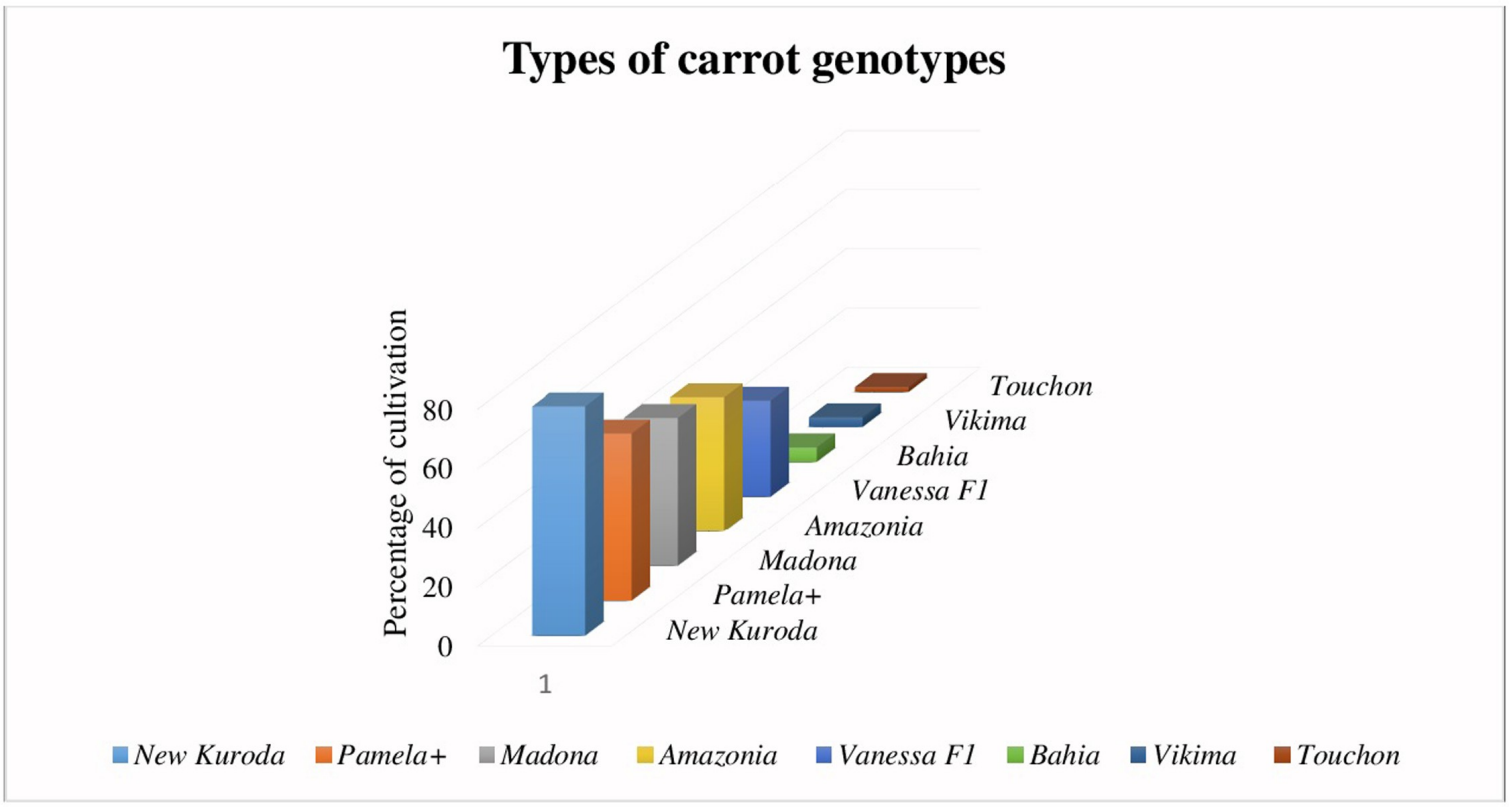
Precentage of distribution of carrot varieties grown in production basins in Cameroon.

**Fig 3:**
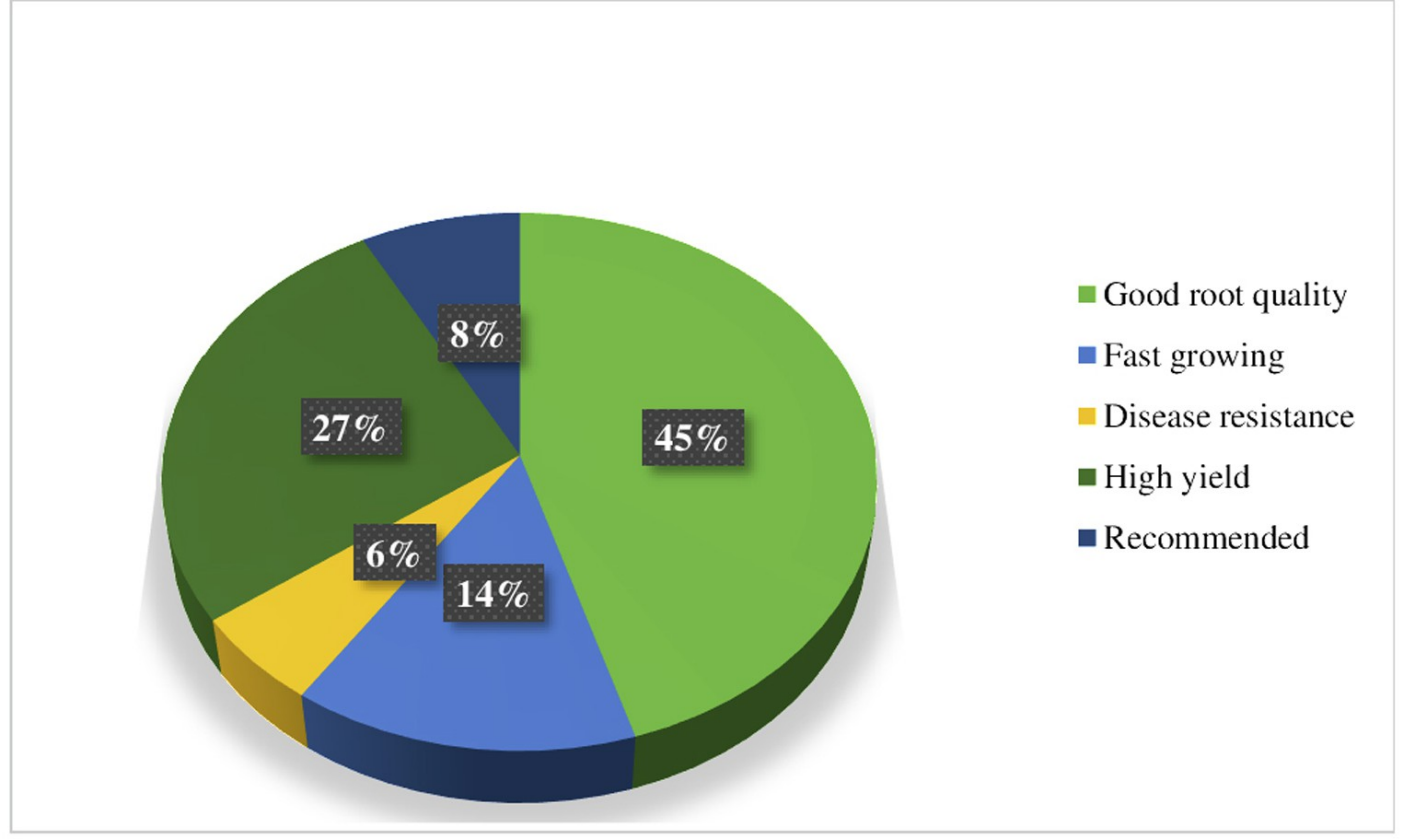
Precentages of the different reasons for the cultivation of carrot varieties declared to be the main varieties grown in Cameroon.

Details on the different factors influencing carrot cultivation in Cameroon and the average yield from the practice are presented in Table 6. From this table, it appears that carrot is grown on slope (90.30%), plain (46.80%) and valley (19.40%). The choice of variety being an indicator of productivity, many farmers use several types of carrot varieties in their production systems for a number of reasons. Five (05) varieties of carrot seeds (Fig 2) were recorded as the most cultivated in the production basins. These include *New Kuroda* (77.40%), *Pamela+* (56.50%), *Madona* (50.00%), *Amazonia* (45.20%) and *Vanessa F1* (32.30%). These carrot varieties are entirely exotic (100%), but are grown for their good root quality (91.90%), profitability (54.80%), fast growth (29.00%) and for recommendation reasons (16.10%) (Fig 3). These carrots are mostly (72.60%) produced twice a year in rotation with other crops either from the same or different crop families.

**Table 6:** Factors influencing carrot productivity.

Characteristics	Frequencies (N = 62)	Percentage (%)
Type of land used		
Summit	2	3.20
Slope	56	90.30
Valley	12	19.40
Plain	29	46.80
Reasons for variety choices		
Good root quality	57	91.90
Fast growing	18	29.00
Disease resistance	7	11.30
High yield	34	54.80
Recommended	10	16.10
Origin of the seeds		
Purchase of imported seeds	62	100
Self-production	0	0
Annual production rate		
1 time	6	9.70
2 times	45	72.60
3 times	11	17.70
Crop rotation		
Yes	62	100.00
No	0	0
Maintenance of plot fertility		
Mineral fertilization	28	45.20
Animal manure through grazing	2	3.20
Crop residue burial	25	40.30
Fallow land	5	8.10
Organic Fertilization	2	3.20
Type of organic fertilizers		
Laying chicken manure	61	98.40
Compost	1	1.60
Amount of organic fertilizers		
≤0.75 t.ha^-1^	8	12.90
0.8–2.5 t.ha^-1^	8	12.90
2.55–5 t.ha^-1^	30	48.40
5.05–7.5 t.ha^-1^	15	24.20
≥7.55 t.ha^-1^	1	1.60
Type of chemical fertilizers		
Urea	15	24.20
NPK : 20_10_10	56	90.30
Amount of chemical fertilizers		
≤250 Kg	21	33.90
300–500 Kg	38	61.30
550–1000 Kg	2	3.20
None	1	1.60
Average yield per plot (Kg)		
˂500	7	11.30
[500–1200]	18	29.00
[1200–2040]	29	46.80
[2040–3000]	8	12.90
High yield season		
Rainy	58	93.50
Dry	4	6.50
Production constraints		
Low germination	36	58.10
High cost of agricultural inputs	34	54.80
Lack of irrigation system	35	56.50
Sales market		
On board field	8	12.90
Local markets	57	91.90
Interurban markets	27	43.50
Marketing constraints		
Overproduction	46	74.20
Transport	44	71.00
Insufficient		
Income in the rainy season/fillet		
3.03 € - 6.06 €	19	30.60
7.57 € - 12.12 €	43	69.40
Income in the dry season/fillet		
7.57 € - 13.63 €	13	21.00
15.14 € - 22.71 €	47	75.80

During the cultivation of carrots in Cameroon, producers use mineral fertilization (45.20%), burying of crop residues (40.30%), fallowing (8.10%) and organic fertilization (3.20%) to maintain the fertility of their agricultural plots. Chicken manure is the main type of organic fertilizer used (98.40%) and NPK 20-10-10 formulation the main type of chemical fertilizer (90.30%). Knowledge of the different types of fertilizers and their quantities applied in the field during carrot production was one of the key aspects of this survey. The quantity of chicken manure used in most cases ranges from 2.55–5 t.ha^-1^ (48.40%) and 5.05–7.5 t.ha^-1^ (24.20%). With regard to the quantities of chemical fertilizers applied, it is generally noted that an average of 300 Kg.ha^-1^ to 500 Kg.ha^-1^ (61.30%) are administered as was solely decided by the farmer. The largest average yield range per plot recorded was between [1200–2040] (46.80%) and this in the rainy season (93.50%) (Table 6).

### 3.5. Constraints

The different constraints faced by carrot producers in Cameroon are presented in Table 6. Farmers often face numerous difficulties at both the production and selling levels. Constraints to carrot production included low germination (58.10%), the lack of efficient irrigation systems (56.50%) and the high costs of agricultural inputs (54.60%).

Constraints to selling included overproduction (74.20%) and the road inaccessibility of most plantation fields which makes transport costs high (71.00%). It is important to note that sales of crop products are mostly made in local markets (91.90%), in interurban markets (43.50%) and at the field (12.90%). The price of these harvested products varies according to the region, the production season and the place of sale. In the rainy season, the price fluctuates between 7.57 € and 12.12 € (69.40%), while in the dry season, it varies between 15.14 € and 22.71 € (75.80%).

Table 7 presents the Chi-square values obtained after analyzing the existence of dependence or not between the variables that influence the carrot farming system. From this table, it emerges that variables such as workforce size, cropping experience, parallel profession, type of organic fertilizer and amount of chemical fertilizer are significantly (p≤ 0.05) associated with farmer’s age. Gender is significantly (p≤ 0.01) associated with variables such as agricultural training and average production yield. There is a highly significant (p≤ 0.001) association between family size and workforce size; cultivated land area and average production yield; amount of organic fertilizer and average production yield. It also exists a significant (p≤ 0.001) relation between the type of organic fertilizer and the amount of chemical fertilizer applied during carrot cultivation.

**Table 7:** Chi-square test values showing the existence of significant dependence or not between the variables affecting the carrot growing system.

Variables	Age	Gender	Education level	Family size	Agricultural training	Land area (ha)	Workforce Size	Experience (year)	Parallel profession	Annual production rate	Type of organic fertilizers	Type of chemical fertilizers	Amount of organic fertilizers	Amount of chemical fertilizers	Average yield per plot
Gender	28.298														
Education level	59.781	4.806													
Family size	114.472	2.119	7.393												
Agricultural training	24.325	7.348^**^	8.029^*^	2.913											
Land area (ha)	74.843	5.904	6.155	17.226	3.594										
Workforce Size	154.576^***^	8.941	7.599	44.096^***^	3.672	25.380^*^									
Experience (year)	139.171^**^	7.845	11.084	29.762^*^	2.890	23.062^*^	39.217^**^								
Parallel profession	127.096^*^	3.485	24.245^**^	20.018	4.656	13.994	28.906^*^	24.562							
Annual production rate	60.660	2.491	2.934	20.148^*^	3.601	6.116	13.208	10.478	10.121						
Type of organic fertilizers	62.000^***^	0.324	4.235	1.234	1.318	4.712	14.738^**^	2.134	6.861	4.712					
Type of chemical fertilizers	33.780	0.303	1.111	1.884	0.282	11.114^*^	7.220	19.979^**^	16.688^**^	4.312	9.486^**^				
Amount of organic fertilizers	95.999	7.748	8.507	10.776	8.543	30.948^**^	18.037	26.774^*^	17.377	7.777	6.861	5.179			
Amount of chemical fertilizers	101.257^*^	2.118	6.543	11.642	3.319	17.025^*^	23.855^*^	26.659^**^	24.151^*^	16.708^*^	62.000^***^	10.905^*^	28.369^**^		
Average yield per plot	65.640	12.965^**^	6.010	8.226	3.521	41.400^***^	17.577	14.435	13.756	5.438	2.485	5.555	41.489^***^	16.292	
Production constraints	18.683	1.056	5.576	1.044	0.028	1.117	3.028	1.726	5.982	1.883	1.407	1.669	3.786	1.566	8.510^*^

^*^ Association is significant at p< 0.05

^**^ Association is significant at p< 0.01

^***^ Association is significant at p< 0.001

## 4. Discussion

The results obtained in this study showed that carrot sales in Cameroon is mainly carried out by young women. This strong involvement of women in carrot sales can be explained by the fact that women are more likely to be unemployed, they turn to the trade of high-profit products to make money in order to meet their daily needs. According to [30], the youth unemployment rate already exceeded 12% in developing countries and is higher in urban than in rural areas, depending on gender and affecting more women than men. Moreover, women are only marginally involved in carrot production and prefer to retail the harvested products. The women’s role in the agricultural production chain and their participation as wholesalers in agricultural trade seems to be low since they are more often retailers than wholesalers [31].

The results also showed that the majority of traders do not have any knowledge about carrot cultivation, including the names of the carrot varieties supplied to them. In fact, out of a list of more than 10 carrot varieties, only two of these carrot varieties (*Pamela* and *New Kuroda*) were known by these traders. The reason that traders do not know the names of carrot varieties could be that they are mostly not in direct contact with farmers to follow the production route and therefore do not have access to production information.

The rotting of carrot roots was recorded as the major constraint in the sale of carrots and could be due to the fact that once the carrots are delivered to these sellers, they resort to inadequate storage methods that encourage product deterioration. Furthermore, this rotting could be accentuated with a long storage period due to the abundance of carrots on the market. Indeed, weight loss as well as diseases are the main causes of post-harvest losses during storage and selling [32].

The results of this survey also showed that the majority of carrot farmers are from the West region, reflecting the existence of several carrot production areas in this region. Such result could be explained by the fact that people in this region are more involved in farming (the main activity in the western region). The objective of this kind of investment of the farmers from the western region in agriculture is to supply the major cities with agricultural products and to earn a substantial income to meet their basic needs. Among these farmers, the fact that the male gender is predominantly involved in carrot cultivation in Cameroon could be a result of men’s great mastery of vegetable cultivation and their ability to recruit employees for large-scale production. In addition, it could be said that this kind of activity requires physical strength to perform. Indeed, the results obtained in this study are similar to those of [33] in Benin who found that the population class exploiting the market gardening domain was mostly represented by men. The latter most often use a labour force made up of mostly young volunteers who practice this activity in order to save money for their daily needs and other life objectives. This is done in order to increase the number of plots to be cultivated, which in turn would increase productivity and thus increase income. In fact, farmers are assisted during vegetable cultivation by permanent or casual labourers who are mostly young people and who engage in this activity to support themselves [34, 35]. However, some young people practice it temporarily in order to have resources to finance their main activities such as studies, trade, etc. [33].

Carrots seem to be mainly grown on sloping land and a large number of producers interviewed are located in the Western region of the country. However, these plots are on slopes as a result of the rugged nature of the land in the Western Region of Cameroon. The possession of exploitable land in the agricultural domain is an advantage that every farmer should enjoy in order to increase his income through the increase of crop products. This study revealed that five main carrot varieties are grown in Cameroon, among which *New Kuroda* and *Pamela+* are the most grown. These high percentages of cultivation by farmers could be attributed to the better nutritional, sensory and agronomic characteristics of these carrot varieties. The information obtained after this survey showed that the area and parcels of land exploited by farmers are significantly correlated with the production yield that the latter declared to obtain at the end of production. Such results could be explained by the fact that the larger is the area, the more the possibilities to perform a large number of plots for cultivation exist and consequently the higher the chances to achieve a high production yield.

The amount of fertilizer applied were also significantly correlated with the yield. This observation could be explained by the fact that fertilization has a great impact on the chemical composition of the cultivated soil by providing nutrients that are assimilated by the crops for their development. Indeed, carrot farmers use fertilizers as a major source of soil nutrients in most cases to achieve high yields [36, 37]. Our previous study showed that fertilization increases production yield [10]. In this present study, no significant relationship was found between the age of the farmer and the yield. This means that the age of the farmer does not determine his or her experience in growing carrots for high productivity. Moreover, when a farmer is advanced in age, he/she might remain in the practice of old cultivation methods and be skeptical to accept new agricultural techniques that could increase product yields. In fact, studies have shown that older people are more reluctant to new ideas and less inclined towards agricultural innovations than younger people [38, 39]. Additionally, the size of the labour force seems to not significantly be related to production output in the sense that whatever the number of people engaged in cultivation in relation to the area under cultivation, the employer’s objective must be met. Furthermore, the size of the workforce could have a negative influence on the time to deliver the desired area and not on the production yield.

The results of this study showed that carrot production is most often reduced because of the low germination rate observed during cultivation, the high purchase prices of agricultural inputs and the lack of irrigation systems during the dry season. Such results could be due to the soil’s lack of nutrients, insufficient knowledge of sowing and irrigation techniques, and farmers’ low incomes. These in turn lead to low productivity, which prevents farmers from having access to all the equipment and agricultural inputs they need to increase their yields. In addition, we have also recorded in this study that certain factors such as overproduction, high transport prices from growing locations to selling towns, are additional factors hindering carrot production in Cameroon. In fact, in Cameroon, most crops are grown in rural areas where access is difficult during the main growing season (rainy season). Our results are similar to those found by Pandey et al. [40] in Nepal who reported at the end of their study that farmers faced production problems such as lack of improved carrot varieties, chemical fertilizers and post-production conservation.

## 5. Conclusion

This study has shown that carrot trading in Cameroonian markets is mainly carried out by women, most of whom come from the West region of Cameroon. Six carrot production basins, namely Dschang, Bamenda, Babadjou, Bafou, Santa and Bamock are best known by the sellers who are also mostly women. Carrot cultivation is mainly carried out by men; they mostly originate from the West region of the country. Fertilization is done by laying chicken manure and chemical fertilizer of formulation 20-10-10 (NPK). The most grown variety are *New Kuroda*, *Pamela+*, *Madona*, *Amazonia* and *Vanessa F1*. Considering the monitoring of carrot production system recorded in this study, it is noted that factors such as farmland area, number of plots cultivated, cropping experience, family size and amounts of organic and chemical fertilizer have a significant impact on agroeconomic production yield. Low germination, the lack of efficient irrigation systems and the high costs of agricultural inputs are the main constraints that affect carrot production in Cameroun.
